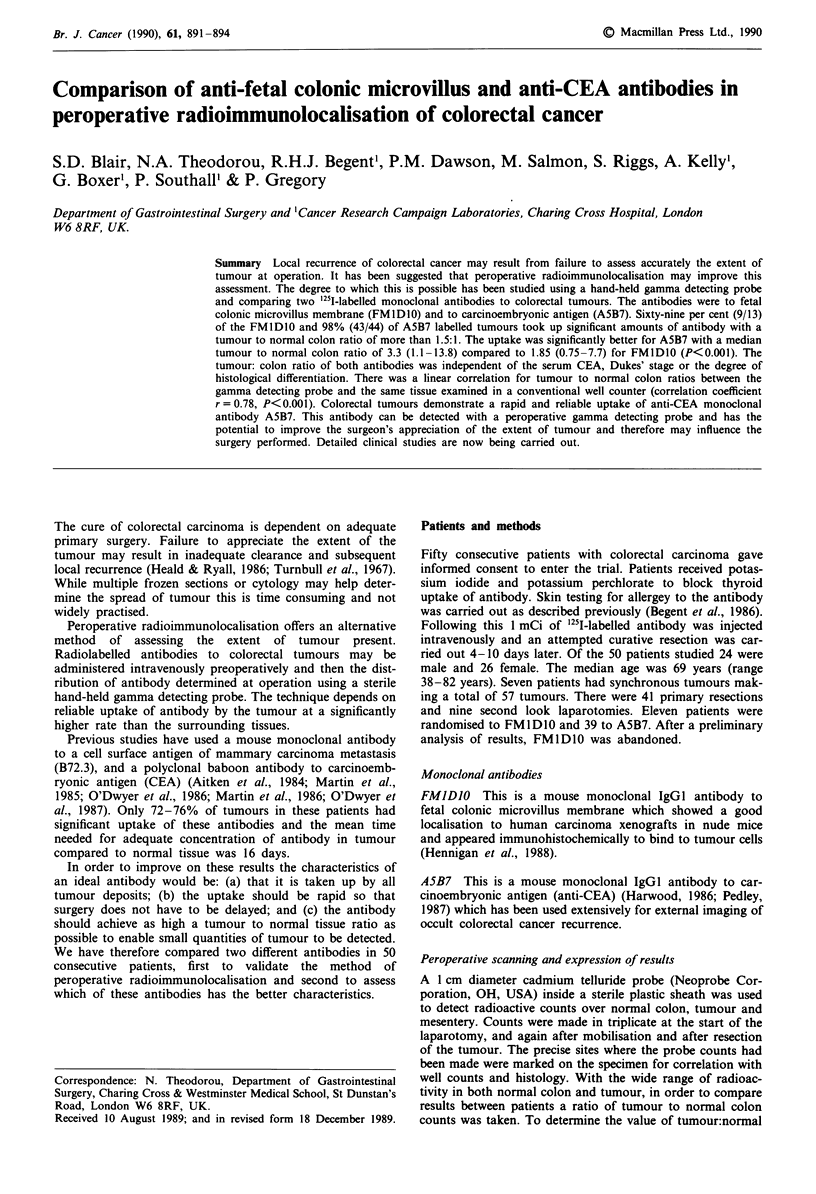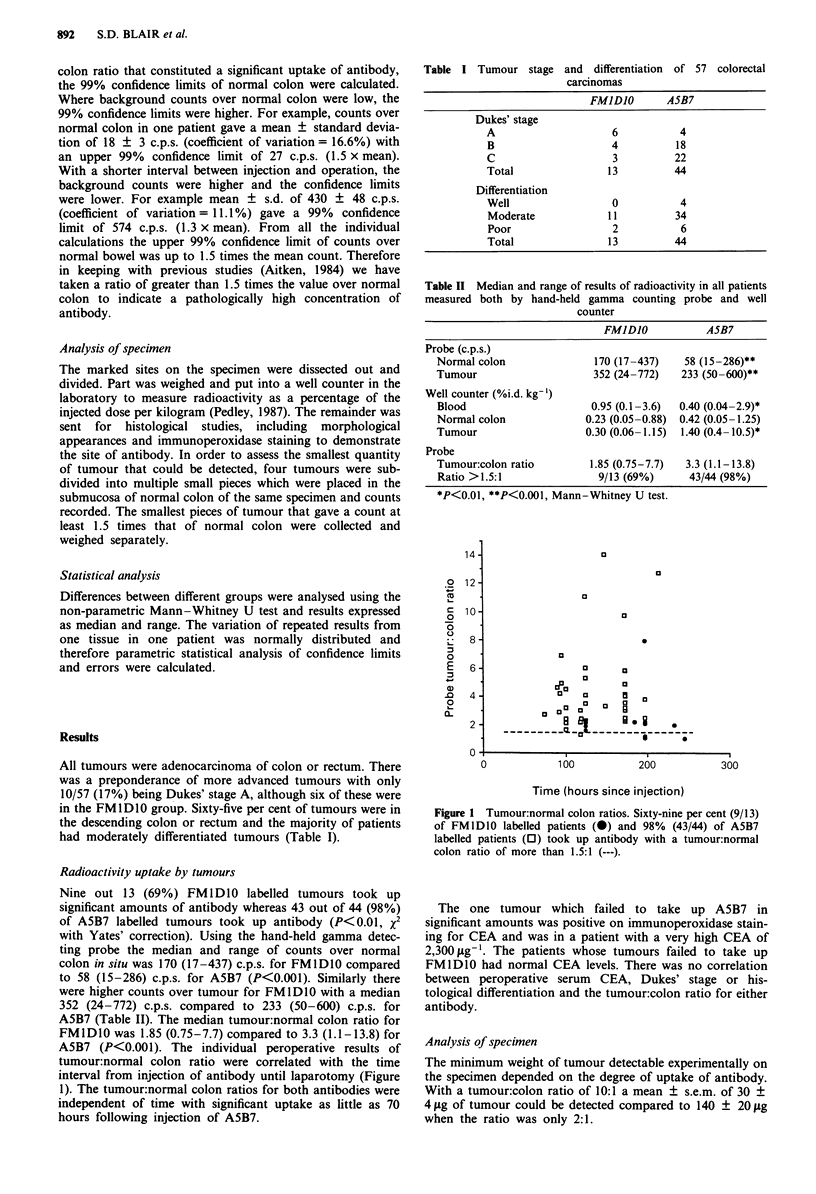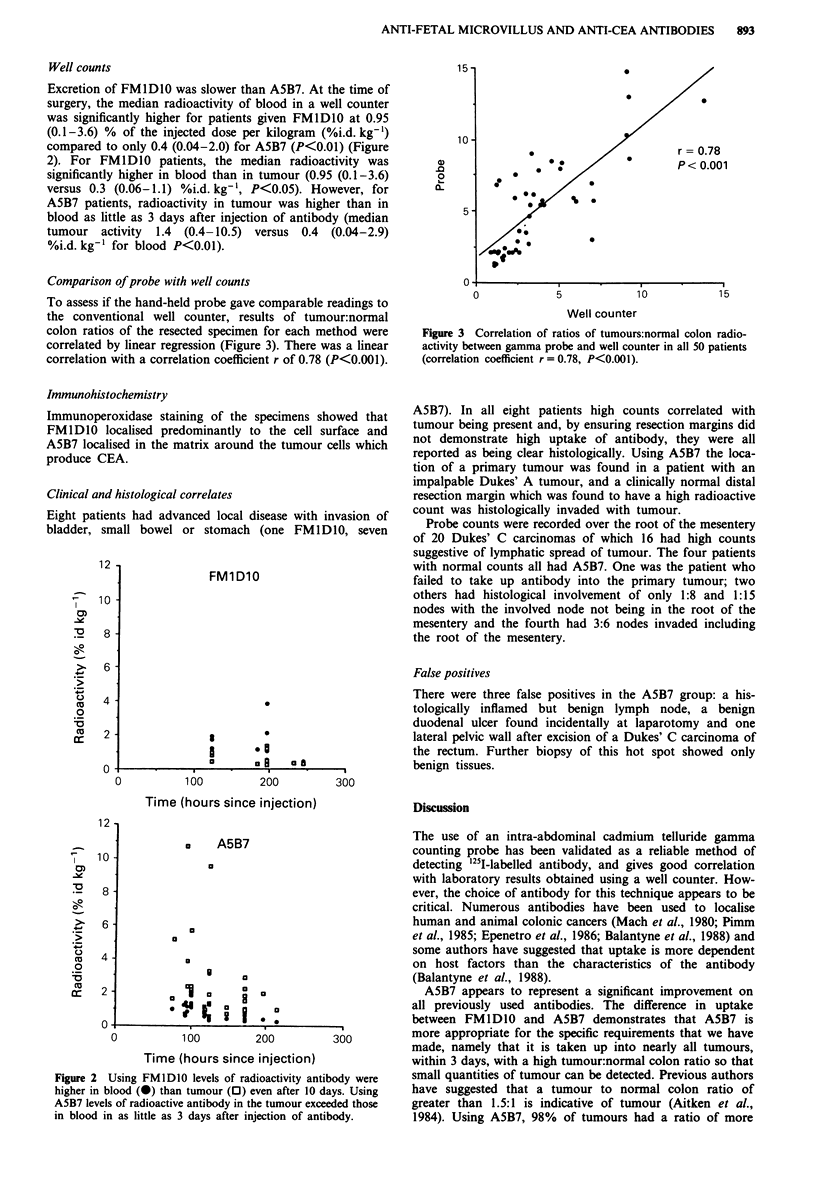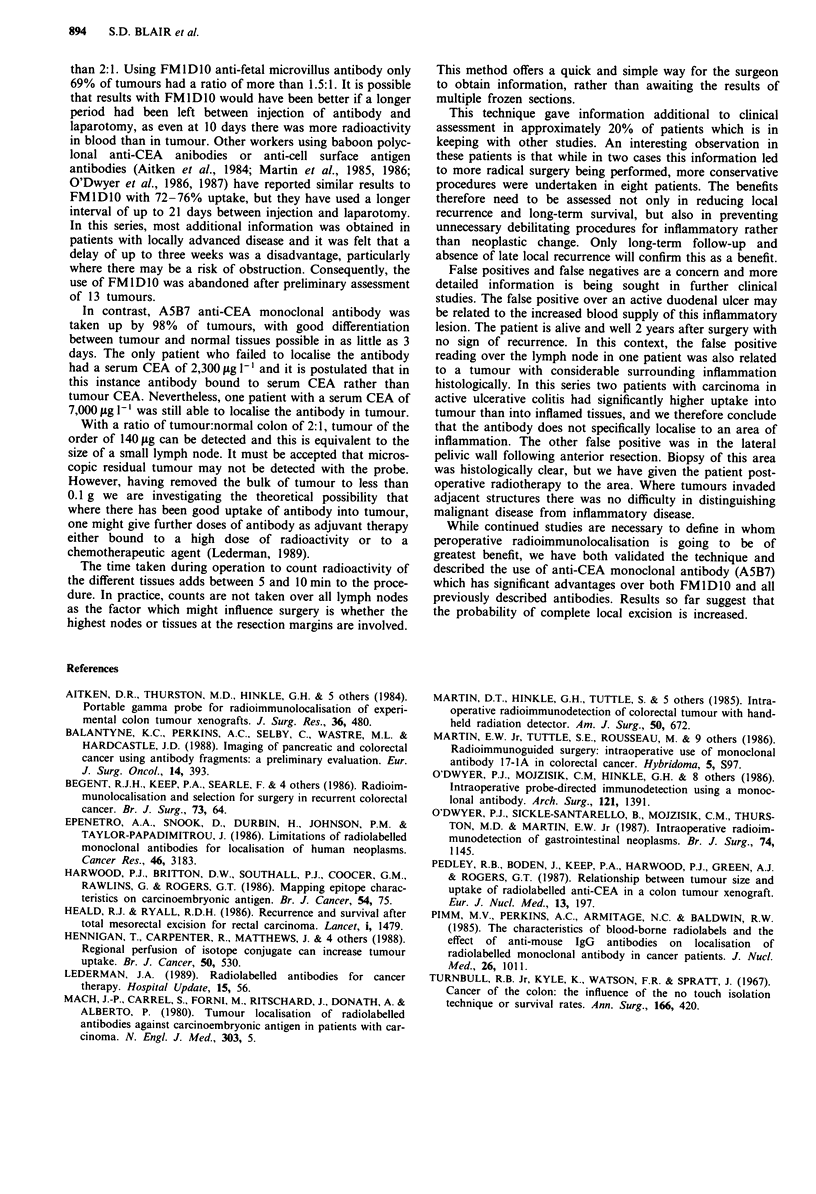# Comparison of anti-fetal colonic microvillus and anti-CEA antibodies in peroperative radioimmunolocalisation of colorectal cancer.

**DOI:** 10.1038/bjc.1990.199

**Published:** 1990-06

**Authors:** S. D. Blair, N. A. Theodorou, R. H. Begent, P. M. Dawson, M. Salmon, S. Riggs, A. Kelly, G. Boxer, P. Southall, P. Gregory

**Affiliations:** Department of Gastrointestinal Surgery, Charing Cross Hospital, London, UK.

## Abstract

Local recurrence of colorectal cancer may result from failure to assess accurately the extent of tumour at operation. It has been suggested that peroperative radioimmunolocalisation may improve this assessment. The degree to which this is possible has been studied using a hand-held gamma detecting probe and comparing two 125I-labelled monoclonal antibodies to colorectal tumours. The antibodies were to fetal colonic microvillus membrane (FM1D10) and to carcinoembryonic antigen (A5B7). Sixty-nine per cent (9/13) of the FM1D10 and 98% (43/44) of A5B7 labelled tumours took up significant amounts of antibody with a tumour to normal colon ratio of more than 1.5:1. The uptake was significantly better for A5B7 with a median tumour to normal colon ratio of 3.3 (1.1-13.8) compared to 1.85 (0.75-7.7) for FM1D10 (P less than 0.001). The tumour: colon ratio of both antibodies was independent of the serum CEA, Dukes' stage or the degree of histological differentiation. There was a linear correlation for tumour to normal colon ratios between the gamma detecting probe and the same tissue examined in a conventional well counter (correlation coefficient r = 0.78, P less than 0.001). Colorectal tumours demonstrate a rapid and reliable uptake of anti-CEA monoclonal antibody A5B7. This antibody can be detected with a peroperative gamma detecting probe and has the potential to improve the surgeon's appreciation of the extent of tumour and therefore may influence the surgery performed. Detailed clinical studies are now being carried out.


Br. J. Cancer (1990), 61, 891-894 © Macmillan Press Ltd., 1990

### Comparison of anti-fetal colonic microvillus and anti-CEA antibodies in peroperative radioimmunolocalisation of colorectal cancer

S.D. Blair, N.A. Theodorou, R.H.J. Begent[1], P.M. Dawson, M. Salmon, S. Riggs, A. Kelly[1], G. Boxer[1], P. Southall[1] & P. Gregory

*Department of Gastrointestinal Surgery and [1]Cancer Research Campaign Laboratories, Charing Cross Hospital, London W6 8RF, UK.*

**Summary** Local recurrence of colorectal cancer may result from failure to assess accurately the extent of tumour at operation. It has been suggested that peroperative radioimmunolocalisation may improve this assessment. The degree to which this is possible has been studied using a hand-held gamma detecting probe and comparing two $^{125}$I-labelled monoclonal antibodies to colorectal tumours. The antibodies were to fetal colonic microvillus membrane (FM1D10) and to carcinoembryonic antigen (A5B7). Sixty-nine per cent (9/13) of the FM1D10 and 98% (43/44) of A5B7 labelled tumours took up significant amounts of antibody with a tumour to normal colon ratio of more than 1.5:1. The uptake was significantly better for A5B7 with a median tumour to normal colon ratio of 3.3 (1.1-13.8) compared to 1.85 (0.75-7.7) for FM1D10 ($P<0.001$). The tumour: colon ratio of both antibodies was independent of the serum CEA, Dukes' stage or the degree of histological differentiation. There was a linear correlation for tumour to normal colon ratios between the gamma detecting probe and the same tissue examined in a conventional well counter (correlation coefficient $r = 0.78$, $P<0.001$). Colorectal tumours demonstrate a rapid and reliable uptake of anti-CEA monoclonal antibody A5B7. This antibody can be detected with a peroperative gamma detecting probe and has the potential to improve the surgeon's appreciation of the extent of tumour and therefore may influence the surgery performed. Detailed clinical studies are now being carried out.

The cure of colorectal carcinoma is dependent on adequate primary surgery. Failure to appreciate the extent of the tumour may result in inadequate clearance and subsequent local recurrence (Heald & Ryall, 1986; Turnbull *et al.*, 1967). While multiple frozen sections or cytology may help determine the spread of tumour this is time consuming and not widely practised.

Peroperative radioimmunolocalisation offers an alternative method of assessing the extent of tumour present. Radiolabelled antibodies to colorectal tumours may be administered intravenously preoperatively and then the distribution of antibody determined at operation using a sterile hand-held gamma detecting probe. The technique depends on reliable uptake of antibody by the tumour at a significantly higher rate than the surrounding tissues.

Previous studies have used a mouse monoclonal antibody to a cell surface antigen of mammary carcinoma metastasis (B72.3), and a polyclonal baboon antibody to carcinoembryonic antigen (CEA) (Aitken *et al.*, 1984; Martin *et al.*, 1985; O'Dwyer *et al.*, 1986; Martin *et al.*, 1986; O'Dwyer *et al.*, 1987). Only 72-76% of tumours in these patients had significant uptake of these antibodies and the mean time needed for adequate concentration of antibody in tumour compared to normal tissue was 16 days.

In order to improve on these results the characteristics of an ideal antibody would be: (a) that it is taken up by all tumour deposits; (b) the uptake should be rapid so that surgery does not have to be delayed; and (c) the antibody should achieve as high a tumour to normal tissue ratio as possible to enable small quantities of tumour to be detected. We have therefore compared two different antibodies in 50 consecutive patients, first to validate the method of peroperative radioimmunolocalisation and second to assess which of these antibodies has the better characteristics.

Correspondence: N. Theodorou, Department of Gastrointestinal Surgery, Charing Cross & Westminster Medical School, St Dunstan's Road, London W6 8RF, UK.
Received 10 August 1989; and in revised form 18 December 1989.

### Patients and methods

Fifty consecutive patients with colorectal carcinoma gave informed consent to enter the trial. Patients received potassium iodide and potassium perchlorate to block thyroid uptake of antibody. Skin testing for allergey to the antibody was carried out as described previously (Begent *et al.*, 1986). Following this 1 mCi of $^{125}$I-labelled antibody was injected intravenously and an attempted curative resection was carried out 4-10 days later. Of the 50 patients studied 24 were male and 26 female. The median age was 69 years (range 38-82 years). Seven patients had synchronous tumours making a total of 57 tumours. There were 41 primary resections and nine second look laparotomies. Eleven patients were randomised to FM1D10 and 39 to A5B7. After a preliminary analysis of results, FM1D10 was abandoned.

### *Monoclonal antibodies*

*FM1D10* This is a mouse monoclonal IgG1 antibody to fetal colonic microvillus membrane which showed a good localisation to human carcinoma xenografts in nude mice and appeared immunohistochemically to bind to tumour cells (Hennigan *et al.*, 1988).

*A5B7* This is a mouse monoclonal IgG1 antibody to carcinoembryonic antigen (anti-CEA) (Harwood, 1986; Pedley, 1987) which has been used extensively for external imaging of occult colorectal cancer recurrence.

### *Peroperative scanning and expression of results*

A 1 cm diameter cadmium telluride probe (Neoprobe Corporation, OH, USA) inside a sterile plastic sheath was used to detect radioactive counts over normal colon, tumour and mesentery. Counts were made in triplicate at the start of the laparotomy, and again after mobilisation and after resection of the tumour. The precise sites where the probe counts had been made were marked on the specimen for correlation with well counts and histology. With the wide range of radioactivity in both normal colon and tumour, in order to compare results between patients a ratio of tumour to normal colon counts was taken. To determine the value of tumour:normal colon ratio that constituted a significant uptake of antibody, the 99% confidence limits of normal colon were calculated. Where background counts over normal colon were low, the 99% confidence limits were higher. For example, counts over normal colon in one patient gave a mean ± standard deviation of 18 ± 3 c.p.s. (coefficient of variation = 16.6%) with an upper 99% confidence limit of 27 c.p.s. (1.5 × mean). With a shorter interval between injection and operation, the background counts were higher and the confidence limits were lower. For example mean ± s.d. of 430 ± 48 c.p.s. (coefficient of variation = 11.1%) gave a 99% confidence limit of 574 c.p.s. (1.3 × mean). From all the individual calculations the upper 99% confidence limit of counts over normal bowel was up to 1.5 times the mean count. Therefore in keeping with previous studies (Aitken, 1984) we have taken a ratio of greater than 1.5 times the value over normal colon to indicate a pathologically high concentration of antibody.

*Analysis of specimen*

The marked sites on the specimen were dissected out and divided. Part was weighed and put into a well counter in the laboratory to measure radioactivity as a percentage of the injected dose per kilogram (Pedley, 1987). The remainder was sent for histological studies, including morphological appearances and immunoperoxidase staining to demonstrate the site of antibody. In order to assess the smallest quantity of tumour that could be detected, four tumours were subdivided into multiple small pieces which were placed in the submucosa of normal colon of the same specimen and counts recorded. The smallest pieces of tumour that gave a count at least 1.5 times that of normal colon were collected and weighed separately.

*Statistical analysis*

Differences between different groups were analysed using the non-parametric Mann–Whitney U test and results expressed as median and range. The variation of repeated results from one tissue in one patient was normally distributed and therefore parametric statistical analysis of confidence limits and errors were calculated.

### Results

All tumours were adenocarcinoma of colon or rectum. There was a preponderance of more advanced tumours with only 10/57 (17%) being Dukes' stage A, although six of these were in the FM1D10 group. Sixty-five per cent of tumours were in the descending colon or rectum and the majority of patients had moderately differentiated tumours (Table I).

*Radioactivity uptake by tumours*

Nine out 13 (69%) FM1D10 labelled tumours took up significant amounts of antibody whereas 43 out of 44 (98%) of A5B7 labelled tumours took up antibody ($P<0.01$, $\chi^2$ with Yates' correction). Using the hand-held gamma detecting probe the median and range of counts over normal colon *in situ* was 170 (17–437) c.p.s. for FM1D10 compared to 58 (15–286) c.p.s. for A5B7 ($P<0.001$). Similarly there were higher counts over tumour for FM1D10 with a median 352 (24–772) c.p.s. compared to 233 (50–600) c.p.s. for A5B7 (Table II). The median tumour:normal colon ratio for FM1D10 was 1.85 (0.75–7.7) compared to 3.3 (1.1–13.8) for A5B7 ($P<0.001$). The individual peroperative results of tumour:normal colon ratio were correlated with the time interval from injection of antibody until laparotomy (Figure 1). The tumour:normal colon ratios for both antibodies were independent of time with significant uptake as little as 70 hours following injection of A5B7.

**Table I** Tumour stage and differentiation of 57 colorectal carcinomas

| | *FM1D10* | *A5B7* |
|---|---|---|
| Dukes' stage | | |
| A | 6 | 4 |
| B | 4 | 18 |
| C | 3 | 22 |
| Total | 13 | 44 |
| Differentiation | | |
| Well | 0 | 4 |
| Moderate | 11 | 34 |
| Poor | 2 | 6 |
| Total | 13 | 44 |

**Table II** Median and range of results of radioactivity in all patients measured both by hand-held gamma counting probe and well counter

| | *FM1D10* | *A5B7* |
|---|---|---|
| Probe (c.p.s.) | | |
| Normal colon | 170 (17–437) | 58 (15–286)** |
| Tumour | 352 (24–772) | 233 (50–600)** |
| Well counter (%i.d. kg$^{-1}$) | | |
| Blood | 0.95 (0.1–3.6) | 0.40 (0.04–2.9)* |
| Normal colon | 0.23 (0.05–0.88) | 0.42 (0.05–1.25) |
| Tumour | 0.30 (0.06–1.15) | 1.40 (0.4–10.5)* |
| Probe | | |
| Tumour:colon ratio | 1.85 (0.75–7.7) | 3.3 (1.1–13.8) |
| Ratio >1.5:1 | 9/13 (69%) | 43/44 (98%) |

*$P<0.01$, **$P<0.001$, Mann–Whitney U test.

**Figure 1** Tumour:normal colon ratios. Sixty-nine per cent (9/13) of FM1D10 labelled patients (●) and 98% (43/44) of A5B7 labelled patients (□) took up antibody with a tumour:normal colon ratio of more than 1.5:1 (---).

The one tumour which failed to take up A5B7 in significant amounts was positive on immunoperoxidase staining for CEA and was in a patient with a very high CEA of 2,300 $\mu g^{-1}$. The patients whose tumours failed to take up FM1D10 had normal CEA levels. There was no correlation between peroperative serum CEA, Dukes' stage or histological differentiation and the tumour:colon ratio for either antibody.

*Analysis of specimen*

The minimum weight of tumour detectable experimentally on the specimen depended on the degree of uptake of antibody. With a tumour:colon ratio of 10:1 a mean ± s.e.m. of 30 ± 4 $\mu$g of tumour could be detected compared to 140 ± 20 $\mu$g when the ratio was only 2:1.

*Well counts*

Excretion of FM1D10 was slower than A5B7. At the time of surgery, the median radioactivity of blood in a well counter was significantly higher for patients given FM1D10 at 0.95 (0.1–3.6) % of the injected dose per kilogram (%i.d. $kg^{-1}$) compared to only 0.4 (0.04–2.0) for A5B7 ($P<0.01$) (Figure 2). For FM1D10 patients, the median radioactivity was significantly higher in blood than in tumour (0.95 (0.1–3.6) versus 0.3 (0.06–1.1) %i.d. $kg^{-1}$, $P<0.05$). However, for A5B7 patients, radioactivity in tumour was higher than in blood as little as 3 days after injection of antibody (median tumour activity 1.4 (0.4–10.5) versus 0.4 (0.04–2.9) %i.d. $kg^{-1}$ for blood $P<0.01$).

*Comparison of probe with well counts*

To assess if the hand-held probe gave comparable readings to the conventional well counter, results of tumour:normal colon ratios of the resected specimen for each method were correlated by linear regression (Figure 3). There was a linear correlation with a correlation coefficient $r$ of 0.78 ($P<0.001$).

**Figure 3** Correlation of ratios of tumours:normal colon radioactivity between gamma probe and well counter in all 50 patients (correlation coefficient $r = 0.78$, $P<0.001$).

*Immunohistochemistry*

Immunoperoxidase staining of the specimens showed that FM1D10 localised predominantly to the cell surface and A5B7 localised in the matrix around the tumour cells which produce CEA.

*Clinical and histological correlates*

Eight patients had advanced local disease with invasion of bladder, small bowel or stomach (one FM1D10, seven A5B7). In all eight patients high counts correlated with tumour being present and, by ensuring resection margins did not demonstrate high uptake of antibody, they were all reported as being clear histologically. Using A5B7 the location of a primary tumour was found in a patient with an impalpable Dukes' A tumour, and a clinically normal distal resection margin which was found to have a high radioactive count was histologically invaded with tumour.

Probe counts were recorded over the root of the mesentery of 20 Dukes' C carcinomas of which 16 had high counts suggestive of lymphatic spread of tumour. The four patients with normal counts all had A5B7. One was the patient who failed to take up antibody into the primary tumour; two others had histological involvement of only 1:8 and 1:15 nodes with the involved node not being in the root of the mesentery and the fourth had 3:6 nodes invaded including the root of the mesentery.

**Figure 2** Using FM1D10 levels of radioactivity antibody were higher in blood (●) than tumour (□) even after 10 days. Using A5B7 levels of radioactive antibody in the tumour exceeded those in blood in as little as 3 days after injection of antibody.

*False positives*

There were three false positives in the A5B7 group: a histologically inflamed but benign lymph node, a benign duodenal ulcer found incidentally at laparotomy and one lateral pelvic wall after excision of a Dukes' C carcinoma of the rectum. Further biopsy of this hot spot showed only benign tissues.

### Discussion

The use of an intra-abdominal cadmium telluride gamma counting probe has been validated as a reliable method of detecting $^{125}$I-labelled antibody, and gives good correlation with laboratory results obtained using a well counter. However, the choice of antibody for this technique appears to be critical. Numerous antibodies have been used to localise human and animal colonic cancers (Mach *et al.*, 1980; Pimm *et al.*, 1985; Epenetro *et al.*, 1986; Balantyne *et al.*, 1988) and some authors have suggested that uptake is more dependent on host factors than the characteristics of the antibody (Balantyne *et al.*, 1988).

A5B7 appears to represent a significant improvement on all previously used antibodies. The difference in uptake between FM1D10 and A5B7 demonstrates that A5B7 is more appropriate for the specific requirements that we have made, namely that it is taken up into nearly all tumours, within 3 days, with a high tumour:normal colon ratio so that small quantities of tumour can be detected. Previous authors have suggested that a tumour to normal colon ratio of greater than 1.5:1 is indicative of tumour (Aitken *et al.*, 1984). Using A5B7, 98% of tumours had a ratio of more than 2:1. Using FM1D10 anti-fetal microvillus antibody only 69% of tumours had a ratio of more than 1.5:1. It is possible that results with FM1D10 would have been better if a longer period had been left between injection of antibody and laparotomy, as even at 10 days there was more radioactivity in blood than in tumour. Other workers using baboon polyclonal anti-CEA anibodies or anti-cell surface antigen antibodies (Aitken *et al.*, 1984; Martin *et al.*, 1985, 1986; O'Dwyer *et al.*, 1986, 1987) have reported similar results to FM1D10 with 72–76% uptake, but they have used a longer interval of up to 21 days between injection and laparotomy. In this series, most additional information was obtained in patients with locally advanced disease and it was felt that a delay of up to three weeks was a disadvantage, particularly where there may be a risk of obstruction. Consequently, the use of FM1D10 was abandoned after preliminary assessment of 13 tumours.

In contrast, A5B7 anti-CEA monoclonal antibody was taken up by 98% of tumours, with good differentiation between tumour and normal tissues possible in as little as 3 days. The only patient who failed to localise the antibody had a serum CEA of 2,300 $\mu g\ l^{-1}$ and it is postulated that in this instance antibody bound to serum CEA rather than tumour CEA. Nevertheless, one patient with a serum CEA of 7,000 $\mu g\ l^{-1}$ was still able to localise the antibody in tumour.

With a ratio of tumour:normal colon of 2:1, tumour of the order of 140 µg can be detected and this is equivalent to the size of a small lymph node. It must be accepted that microscopic residual tumour may not be detected with the probe. However, having removed the bulk of tumour to less than 0.1 g we are investigating the theoretical possibility that where there has been good uptake of antibody into tumour, one might give further doses of antibody as adjuvant therapy either bound to a high dose of radioactivity or to a chemotherapeutic agent (Lederman, 1989).

The time taken during operation to count radioactivity of the different tissues adds between 5 and 10 min to the procedure. In practice, counts are not taken over all lymph nodes as the factor which might influence surgery is whether the highest nodes or tissues at the resection margins are involved. This method offers a quick and simple way for the surgeon to obtain information, rather than awaiting the results of multiple frozen sections.

This technique gave information additional to clinical assessment in approximately 20% of patients which is in keeping with other studies. An interesting observation in these patients is that while in two cases this information led to more radical surgery being performed, more conservative procedures were undertaken in eight patients. The benefits therefore need to be assessed not only in reducing local recurrence and long-term survival, but also in preventing unnecessary debilitating procedures for inflammatory rather than neoplastic change. Only long-term follow-up and absence of late local recurrence will confirm this as a benefit.

False positives and false negatives are a concern and more detailed information is being sought in further clinical studies. The false positive over an active duodenal ulcer may be related to the increased blood supply of this inflammatory lesion. The patient is alive and well 2 years after surgery with no sign of recurrence. In this context, the false positive reading over the lymph node in one patient was also related to a tumour with considerable surrounding inflammation histologically. In this series two patients with carcinoma in active ulcerative colitis had significantly higher uptake into tumour than into inflamed tissues, and we therefore conclude that the antibody does not specifically localise to an area of inflammation. The other false positive was in the lateral pelivic wall following anterior resection. Biopsy of this area was histologically clear, but we have given the patient post-operative radiotherapy to the area. Where tumours invaded adjacent structures there was no difficulty in distinguishing malignant disease from inflammatory disease.

While continued studies are necessary to define in whom peroperative radioimmunolocalisation is going to be of greatest benefit, we have both validated the technique and described the use of anti-CEA monoclonal antibody (A5B7) which has significant advantages over both FM1D10 and all previously described antibodies. Results so far suggest that the probability of complete local excision is increased.

### References

AITKEN, D.R., THURSTON, M.D., HINKLE, G.H. & 5 others (1984). Portable gamma probe for radioimmunolocalisation of experimental colon tumour xenografts. *J. Surg. Res.*, **36**, 480.

BALANTYNE, K.C., PERKINS, A.C., SELBY, C., WASTRE, M.L. & HARDCASTLE, J.D. (1988). Imaging of pancreatic and colorectal cancer using antibody fragments: a preliminary evaluation. *Eur. J. Surg. Oncol.*, **14**, 393.

BEGENT, R.J.H., KEEP, P.A., SEARLE, F. & 4 others (1986). Radioimmunolocalisation and selection for surgery in recurrent colorectal cancer. *Br. J. Surg.*, **73**, 64.

EPENETRO, A.A., SNOOK, D., DURBIN, H., JOHNSON, P.M. & TAYLOR-PAPADIMITROU, J. (1986). Limitations of radiolabelled monoclonal antibodies for localisation of human neoplasms. *Cancer Res.*, **46**, 3183.

HARWOOD, P.J., BRITTON, D.W., SOUTHALL, P.J., COOCER, G.M., RAWLINS, G. & ROGERS, G.T. (1986). Mapping epitope characteristics on carcinoembryonic antigen. *Br. J. Cancer*, **54**, 75.

HEALD, R.J. & RYALL, R.D.H. (1986). Recurrence and survival after total mesorectal excision for rectal carcinoma. *Lancet*, **i**, 1479.

HENNIGAN, T., CARPENTER, R., MATTHEWS, J. & 4 others (1988). Regional perfusion of isotope conjugate can increase tumour uptake. *Br. J. Cancer*, **50**, 530.

LEDERMAN, J.A. (1989). Radiolabelled antibodies for cancer therapy. *Hospital Update*, **15**, 56.

MACH, J.-P., CARREL, S., FORNI, M., RITSCHARD, J., DONATH, A. & ALBERTO, P. (1980). Tumour localisation of radiolabelled antibodies against carcinoembryonic antigen in patients with carcinoma. *N. Engl. J. Med.*, **303**, 5.

MARTIN, D.T., HINKLE, G.H., TUTTLE, S. & 5 others (1985). Intraoperative radioimmunodetection of colorectal tumour with hand-held radiation detector. *Am. J. Surg.*, **50**, 672.

MARTIN, E.W. Jr, TUTTLE, S.E., ROUSSEAU, M. & 9 others (1986). Radioimmunoguided surgery: intraoperative use of monoclonal antibody 17-1A in colorectal cancer. *Hybridoma*, **5**, S97.

O'DWYER, P.J., MOJZISIK, C.M, HINKLE, G.H. & 8 others (1986). Intraoperative probe-directed immunodetection using a monoclonal antibody. *Arch. Surg.*, **121**, 1391.

O'DWYER, P.J., SICKLE-SANTARELLO, B., MOJZISIK, C.M., THURSTON, M.D. & MARTIN, E.W. Jr (1987). Intraoperative radioimmunodetection of gastrointestinal neoplasms. *Br. J. Surg.*, **74**, 1145.

PEDLEY, R.B., BODEN, J., KEEP, P.A., HARWOOD, P.J., GREEN, A.J. & ROGERS, G.T. (1987). Relationship between tumour size and uptake of radiolabelled anti-CEA in a colon tumour xenograft. *Eur. J. Nucl. Med.*, **13**, 197.

PIMM, M.V., PERKINS, A.C., ARMITAGE, N.C. & BALDWIN, R.W. (1985). The characteristics of blood-borne radiolabels and the effect of anti-mouse IgG antibodies on localisation of radiolabelled monoclonal antibody in cancer patients. *J. Nucl. Med.*, **26**, 1011.

TURNBULL, R.B. Jr, KYLE, K., WATSON, F.R. & SPRATT, J. (1967). Cancer of the colon: the influence of the no touch isolation technique or survival rates. *Ann. Surg.*, **166**, 420.